# Editorial: Physiological and pathological foundations of female reproductive function

**DOI:** 10.3389/fcell.2026.1963682

**Published:** 2026-08-17

**Authors:** Liling Liu, Yuhua Shi, Xiang-Shun Cui, Heng-Yu Fan

**Affiliations:** 1 Department of Reproductive Medicine and Genetics Center, The People’s Hospital of Guangxi Zhuang Autonomous Region, Nanning, Guangxi, China; 2 Department of Reproductive Medicine, Nanfang Hospital, Southern Medical University, Guangzhou, China; 3 Department of Animal Science, Chungbuk National University, Cheongju, Chungbuk, Republic of Korea; 4 MOE Key Laboratory for Biosystems Homeostasis, Life Sciences Institute, Zhejiang University, Hangzhou, China; 5 Zhejiang Key Laboratory of Precise Protection and Promotion of Fertility, Assisted Reproduction Unit, Department of Obstetrics and Gynecology, Sir Run Run Shaw Hospital, Zhejiang University, Hangzhou, China

**Keywords:** assisted reproduction, early embryogenesis, female reproduction, fertilization, germ cell, infertility

Female reproductive function relies on a precisely orchestrated sequence of molecular, cellular, and microenvironmental events—from oocyte maturation and fertilization to early embryo development and implantation. Disruptions at any of these stages, whether caused by intrinsic genetic factors or extrinsic environmental stressors, can lead to reproductive failure, infertility, or pregnancy complications. Despite significant advances in assisted reproductive technologies (ART) and our understanding of gamete and embryo biology, the molecular underpinnings of many reproductive disorders remain incompletely understood, and clinical outcomes often lag behind expectations. This Research Topic brings together 19 original research articles and reviews that collectively advance our understanding of the physiological and pathological foundations of female reproductive function, spanning from ovarian reserve assessment and oocyte quality to endometrial receptivity, implantation, and early pregnancy maintenance.

A central theme emerging from this Research Topic is the importance of the ovarian and uterine microenvironment in determining reproductive success. Li et al. examined the interaction between ovarian reserve markers—anti-Müllerian hormone (AMH), antral follicle count (AFC), and basal follicle-stimulating hormone (FSH)—and endometrial thickness on ART outcomes in over 12,000 fresh embryo transfer cycles. They found that in patients with normal ovarian reserve (AMH ≥2.59 ng/mL or AFC ≥11), each 1-mm increase in endometrial thickness was associated with modest but significant improvements in biochemical pregnancy, clinical pregnancy, and live birth rates, highlighting the synergistic relationship between ovarian function and endometrial receptivity. Complementing this, Wang et al. focused on POSEIDON Group 1 patients—young women with normal ovarian reserve but unexpected poor response—and demonstrated that serum progesterone levels on the day of embryo transfer exhibit a non-linear association with clinical pregnancy, with an optimal range between the 10th and 90th percentiles (9.45–23.32 ng/mL). Notably, body mass index significantly modified this association: elevated progesterone was detrimental in normal-weight patients but not in overweight/obese individuals, underscoring the need for individualized luteal support strategies.

The follicular fluid microenvironment received particular attention in two complementary studies. Jiang et al. employed integrated proteomic and metabolomic profiling of human follicular fluid from follicles of different diameters, identifying 81 differentially abundant proteins and 141 differentially abundant metabolites between large (>18 mm) and small (<14 mm) follicles. Among the key findings, tubulin α4A (TUBA4A) and tubulin α1C (TUBA1C) were significantly elevated in small follicles and validated by ELISA, implicating microtubule dynamics in early oocyte development. Cai et al. took a different approach, examining preimplantation hematological parameters in 717 frozen embryo transfer cycles. They found that the triglyceride-glucose index (TyG Index) and TyG-BMI—markers of insulin resistance—were significantly associated with reduced clinical pregnancy rates, with TyG-BMI showing incremental predictive value beyond conventional clinical models. Together, these studies underscore the utility of multi-omics approaches and accessible biomarkers for assessing oocyte and embryo quality.

Several studies explored novel therapeutic and diagnostic strategies for challenging reproductive conditions. Xing et al. evaluated a botanical formulation (DACHAO Reco18) containing clove, Sophora flower bud, and yam extracts in 68 IVF patients with prior poor embryo quality. In this prospective self-controlled study, 6 weeks of oral supplementation significantly improved oocyte yield, fertilization rates, and high-quality embryo numbers, with a 118.5% increase in high-quality embryos and a reduction in FSH levels in hypergonadotropic patients. Xing et al. also investigated intrauterine infusion of human umbilical cord blood mononuclear cells (HUCBMCs) in 134 patients with thin endometrium undergoing frozen embryo transfer. While the treatment was safe, no significant improvements in live birth or pregnancy outcomes were observed compared with controls, though blastocyst transfer emerged as a strong positive predictor of live birth, emphasizing the importance of embryo stage in this difficult-to-treat population.

Machine learning and artificial intelligence are increasingly being applied to reproductive medicine, as demonstrated by two studies in this Research Topic. Liu et al. developed an interpretable Extreme Gradient Boosting (XGBoost) model to predict cumulative live birth in 194 women with ovarian endometriomas undergoing ethanol sclerotherapy followed by IVF/ICSI. The model achieved an AUC of 0.830 and identified AFC, progesterone on Gn starting day, downregulation, cyst diameter, and previous live birth history as the five most influential predictors, with SHAP analysis providing transparent, individualized predictions. Hou et al. proposed AutoPCOS, a stepwise multimodal intelligent framework for PCOS risk stratification that integrates clinical, laboratory, and ultrasound features. The framework supports flexible, resource-aware diagnostic pathways—clinical-only, clinical + laboratory, clinical + ultrasound, and full multimodal models—with the full model achieving 94.8% accuracy. Integration with the Lingshu large language model enables interpretable risk explanations and personalized recommendations, bridging the gap between algorithmic performance and clinical utility.

The inflammatory and immune landscape of female reproductive disorders was explored in several contributions. Duan et al. provided a comprehensive review of endogenous mediators in primary dysmenorrhea, synthesizing evidence on prostaglandins, vasopressin, estradiol, oxytocin, endothelin-1, cytokines, and neurotransmitters that contribute to uterine contraction, inflammation, and pain. They highlighted the potential of metabolomics, proteomics, and neuroimaging to identify novel biomarkers for this highly prevalent but understudied condition. Zhang et al. reviewed the role of IL-33/ST2 signaling in female reproductive diseases, proposing that this pathway functions as a context- and stage-dependent regulatory axis rather than a simple pro- or anti-inflammatory mediator. They synthesized evidence across endometriosis, recurrent miscarriage, polycystic ovary syndrome, and primary ovarian insufficiency, emphasizing that therapeutic development should prioritize precise, stage-specific, and cell-selective modulation rather than indiscriminate systemic blockade. Du et al. investigated the molecular basis of oocyte dysfunction in PCOS using a DHEA-induced mouse model. Through parallel Smart-seq2 profiling of GV and MII oocytes, they discovered that PCOS oocytes maintain transcriptomic parity at the GV stage but undergo a profound collapse specifically during the GV-to-MII transition. This maturation-coupled failure was characterized by blunted activation of Lsm14b—a central P-body assembly factor—leading to dual-layer post-transcriptional dysregulation: aberrant retention of mitochondrial OXPHOS transcripts (Sdhb, Cox6a1) and hyper-depletion of oocyte identity genes (Figla, Zp3). These findings reframe PCOS-associated oocyte dysfunction as a stage-specific failure of post-transcriptional remodeling competence and identify the Lsm14b-P-body axis as a candidate therapeutic target.

Finally, Barroso Alverde et al. conducted a systematic review of evidence-based interventions to restore or improve fertility in women aged 30–42 years, synthesizing 21 studies across etiologies including PCOS, endometriosis, poor ovarian response, and recurrent implantation failure. They found that while several hormonal and ART strategies improve pregnancy-related outcomes, evidence for live birth remains limited, and safety reporting is sparse. The review underscores the critical need for adequately powered randomized trials with live birth as the primary endpoint, standardized core outcome sets, and systematic capture of maternal-neonatal safety.

Collectively, the articles in this Research Topic illuminate the complex interplay between molecular regulators, microenvironmental factors, and systemic conditions that determine female reproductive success. They highlight the value of integrating multi-omics approaches, advanced biomarkers, and machine learning to refine diagnosis and personalize treatment. They also reveal persistent gaps—particularly the need for therapies that address the qualitative defects in oocyte and embryo quality, and for interventions validated by live birth and safety outcomes. As we continue to unravel the physiological and pathological foundations of female reproductive function, the contributions gathered here provide a solid foundation for future research and clinical translation, ultimately aiming to improve reproductive health outcomes for women worldwide.

